# Response of Wheat to a Multiple Species Microbial Inoculant Compared to Fertilizer Application

**DOI:** 10.3389/fpls.2018.01601

**Published:** 2018-11-13

**Authors:** Salmabi K. Assainar, Lynette K. Abbott, Bede S. Mickan, Andrew S. Whiteley, Kadambot H. M. Siddique, Zakaria M. Solaiman

**Affiliations:** ^1^UWA School of Agriculture and Environment, The University of Western Australia, Perth, WA, Australia; ^2^The UWA Institute of Agriculture, The University of Western Australia, Perth, WA, Australia; ^3^Richgro Garden Products, Jandakot, WA, Australia

**Keywords:** biostimulants, mineral fertilizer, chemical fertilizer, grain yield, harvest index, abiotic stress

## Abstract

Microbial inoculants, including those formed from multiple species, may have dual functions as biostimulants and/or biocontrol agents, and claimed agricultural benefits are instrumental for regulatory categorisation. Biostimulants include commercial products containing substances or microorganisms that stimulate plant growth. Biostimulant microbes can be involved in a range of processes that affect N and P transformations in soil and thus influence nutrient availability, and N and P fertilizers can influence soil microbial diversity and function. A glasshouse experiment was conducted to investigate the effect of a multiple species microbial inoculant relative to a rock-based mineral fertilizer and a chemical fertilizer on wheat growth and yield, and on microbial diversity in the rhizosphere. The microbial inoculant was compared to the mineral fertilizer (equivalent to 5.6 kg N ha^-1^ and 5.6 kg P ha^-1^), and to the chemical fertilizer applied at three rates equivalent to: (i) 7.3 kg N ha^-1^ and 8.4 kg P ha^-1^ as recommended for on-farm use, (ii) 5.6 kg N ha^-1^ and 6.5 kg P ha^-1^ which matched the N in the mineral fertilizer, and (iii) 4.9 kg N ha^-1^ and 5.6 kg P ha^-1^ which matched P content in the mineral fertilizer. Despite an early reduction in plant growth, the microbial inoculant treatment increased shoot growth at maturity compared to the control. Similarly, grain yield was higher after application of the microbial inoculant when compared to control, and it was similar to that of plants receiving the fertilizer treatments. Using 16S rRNA sequencing, the microbial inoculant and fertilizer treatments were shown to influence the diversity of rhizosphere bacteria. The microbial inoculant increased the relative abundance of the phylum *Actinobacteria*. At tillering, the proportion of roots colonized by arbuscular mycorrhizal (AM) fungi increased with the microbial inoculant and mineral fertilizer treatments, but decreased with the chemical fertilizer treatments. At maturity, there were no treatment effects on the proportion of wheat roots colonized by AM fungi. Overall, the multiple species microbial inoculant had beneficial effects in terms of wheat yield relative to the commercial mineral and chemical fertilizers applied at the level recommended for on-farm use in south-western Australia.

## Introduction

Biostimulants are derived from a wide range of materials including live microbial cultures, extracts of microbes, animal or plant origin, soil organic compounds (humic and fulvic acids), industrial by-products and chemicals, and synthetic molecules (Saa et al., 2015). Biostimulants can stimulate plant growth and yield even when used in small amounts, but not to the same extent as traditional fertilizer (Fleming et al., 2014). Various rhizosphere microorganisms synthesize plant growth-promoters, siderophores and antibiotics, hence, plant-microbe symbioses can reduce dependency on nitrogen (N) and phosphorus (P) fertilizers (Ahemad and Kibret, 2014). Some plant responses to biostimulants cannot be explained by current understanding, and this represents both a challenge and an opportunity (Brown and Saa, 2015). The global market for biostimulants is projected to increase by 12% per year and reach more than US$2.2 billion by 2018 (Calvo et al., 2014).

Soil microbial communities are extremely diverse and perform key functions, including the cycling of carbon (C), nutrients and water, maintaining soil productivity, dissolution of rock minerals, and remediation of contaminants (Schmitz et al., 2015). There is potential to increase the effectiveness of poorly soluble rock phosphate by managing soil biological processes, with some microorganisms contributing to the dissolution of ground rock fertilizers through the release of organic ligands, H^+^ ions and organic acids into the soil (Barker et al., 1997; Hinsinger et al., 2001; Richardson, 2001). In nutrient-deficient soils, microorganisms may preferentially colonize ground rock fertilizers if they contain growth-limiting nutrients. For example, Roger and Bennett (2004) showed that in a P-limiting environment, microorganisms selectively colonized the surface of minerals containing P. The effectiveness of plant access to P in rock phosphate can be improved by the activities of arbuscular mycorrhizal (AM) fungi (Barrow et al., 1977; Pairunan et al., 1980). Soil microorganisms are vital constituents of the rhizosphere, and they play key roles in P cycling (Richardson, 1994). AM fungi are members of the Glomeromycota, a key component of the soil microbiota which form the most common and widespread terrestrial plant symbioses. They are obligate symbiotic soil fungi, and they form intimate associations with approximately 80% of terrestrial plant species including the majority of crops (Smith and Read, 2008). AM fungi have been shown to benefit crop productivity due to their contribution to plant nutrition, soil structure and other ecosystem services (Smith and Read, 2008). The predominant function of AM fungi is attributed to increased host plant P uptake as a consequence of their high-affinity P uptake mechanism (Smith and Smith, 2012). Ground rock addition to soil is likely to affect microbial communities through indirect effects on physicochemical transformations (Hinsinger et al., 1996; Gillmann et al., 2002; Loganathan et al., 2002; Priyono and Gilkes, 2004) and by increasing plant growth (Sanz and Rowell, 1988; Coroneos et al., 1996; Bakken et al., 2000).

The two most-reported mechanisms by which microorganisms solubilise P are the production of organic acids (Goldstein, 1996) and the production of phosphatases (Rodriguez et al., 2006). Organic acids transform insoluble phosphate forms into soluble forms through their hydroxyl groups. These groups chelate the cations bound to phosphate, thereby facilitating the release of phosphate ions (Rodriguez and Fraga, 1999). In nutrient-poor soils, such as the highly weathered soils in south-western Australia, minerals containing limiting concentrations of nutrients may influence the soil microbial community structure (Carson et al., 2012). Phosphate solubilisation takes place through various microbial processes/mechanisms including organic acid production and proton extrusion as a result of the combined effect of a pH decrease and organic acid production (He and Zhu, 1988; Surange et al., 1995; Dutton and Evans, 1996; Nahas, 1996; Deubel and Merbach, 2005; Fankem et al., 2006).

Rhizosphere microorganisms, including plant growth promoting rhizobacteria (PGPRs) have been investigated for their effects on plant growth (Adesemoye et al., 2008). One proposed mechanism by which PGPRs can affect nutrient uptake is by enhancing growth and development of roots, leading to larger root systems with the greater surface area and more root hairs, which are then able to access more nutrients (Biswas et al., 2000a; Adesemoye et al., 2008). These studies have included comparisons with plant-microbe interactions and fertilizer use efficiency (Adesemoye et al., 2008).

We compared the efficacy of a multiple species microbial inoculant to a rock-based mineral fertilizer and a more traditional chemical fertilizer for its capacity to increase wheat yield in an agricultural soil that was moderately deficient in N and P for the growth of wheat. We hypothesized that the microbial inoculant would be as effective as the mineral fertilizer on growth, yield and nutrient uptake of wheat based on its potential to supply N and P through mineralization of organic matter. The microbial inoculant was expected to be less effective than the chemical fertilizer. Mechanisms underlying these hypotheses could be associated with activities of rhizosphere bacterial communities and AM fungi whereby (1) the introduction of the microbial inoculant would augment the existing rhizosphere bacterial community, and (2) the application of the chemical fertilizer would reduce mycorrhizal colonization of wheat roots but the microbial inoculant and mineral fertilizer would not affect colonization.

## Materials and Methods

### Experimental Design

A multiple species microbial inoculant and two fertilizers (a rock-based mineral fertilizer, and a chemical fertilizer) were applied to soil before sowing wheat seeds. A control treatment had neither microbial inoculant nor fertilizer added to the soil which was collected from an agricultural field and was moderately N and P deficient for growing wheat. There were four replicates of each treatment. The experiment was arranged in a completely randomized design. Plants were harvested at tillering (7 weeks) and maturity (12 weeks).

Six treatments were (Table 1): (i) a control that did not receive any amendments; (ii) a multiple species microbial inoculant (1 g powder/pot applied), (iii) MF: a rock-mineral fertilizer at 75 kg ha^-1^ (equivalent to 5.6 kg N ha^-1^ and 5.6 kg P ha^-1^), (iv) CF-1: 75 kg ha^-1^ of a traditional soluble chemical fertilizer (equivalent to 7.3 kg N ha^-1^ and 8.4 kg P ha^-1^, applied at the recommended on-farm), (v) CF-2: 55 kg ha^-1^ of the same chemical fertilizer (equivalent to 5.6 kg N ha^-1^ and 6.5 kg P ha^-1^ that matched the N concentration in the mineral fertilizer), and (vi) CF-3: 43 kg ha^-1^ of the same chemical fertilizer (equivalent to 4.9 kg N ha^-1^ and 5.6 kg P ha^-1^, which matched the P concentration in the mineral fertilizer).

**Table 1:** Composition of treatments applied at the commencement of the experiment.

Treatment	Composition	Application rate
Control	Unamended	Nil
Microbial inoculant (Microbes)	A multiple species microbial inoculant is a talc-based formulation containing (per g) isolates of *Agrobacterium (1 × 10ˆ9), Azotobacter (1.2 × 10ˆ9),* *Azospirillum (1.1 × 10ˆ9), Bacillus (112 × 10ˆ9), Pseudomonas (2.3 × 10ˆ9), Streptomyces (1 × 10ˆ9), Trichoderma (8 × 10ˆ9),* and *Rhizophagus irregularis (75 spores).*	Microbial inoculant was provided by Australian Mineral Fertilizer Pty Ltd. as a powder form and applied at the rate of 1g pot^-1^
Mineral fertilizer (MF)	Mineral-based fertilizer (from Australian Mineral Fertilizer Pty Ltd.), consists of a proprietary combination of fine mineral ores, such as micas, alkali feldspars, soft rock phosphate, dolomite, basalt, granite and crystalline silica, that are blended with various sulfates (ammonium, potassium, manganese, copper and zinc) containing nutrients (in %, w/w) N-7.5, P 7.5, K-4.5, S-8.0, Mg-0.9, Fe-2.6, Si-6.7, Mn-0.4, Zn-0.043, Cu-0.043, B-0.0017	Equivalent to 5.6 kg N and 5.6 kg P ha^-1^; (1g pot^-1^)
Chemical fertilizer -1 75 kg ha^-1^ (CF-1)	Gusto Gold from Summit Fertilizers - fully granulated compound fertilizer with all nutrients in each granule as in % (w/w) N-10.2, P-13.1, K-12.0, S -7.2, Cu-0.09, Zn-0.13	Equivalent to 7.3 kg N and 8.4 kg P ha^-1^ which includes recommended on-farm equivalent rates of N and P respectively; (1g pot^-1^)
Chemical fertilizer - 2 55 kg ha^-1^ (CF-2)	Gusto Gold from Summit Fertilizers- fully granulated compound fertilizer with all nutrients in each granule as in % (w/w) N-10.2, P-13.1, K-12.0, S -7.2, Cu-0.09, Zn-0.13	Equivalent to 5.6 kg N and 6.5 kg P ha^-1^ which matched the N in the mineral fertilizer; (0.735g pot^-1^)
Chemical fertilizer – 3 43 kg ha^-1^ (CF-3)	Gusto Gold – Summit Fertilizers- fully granulated compound fertilizer with all nutrients in each granule as in % (w/w) N-10.2, P-13.1, K-12.0, S -7.2, Cu-0.09, Zn-0.13	Equivalent to 4.9 kg N and 5.6 kg P ha^-1^ which matched the P in the mineral fertilizer; (0.574g pot^-1^)


### Soil Properties and Plant Growth Conditions

Field soil at 0–10 cm depth was collected from an agricultural field at Dowerin, Western Australia (latitude 31.22^0^S, longitude 117.02^0^E). The soil was a moderately nutrient-deficient, loamy sand with the following properties: pH (1:5, soil/water) 5.7, EC: 0.133 dS m^-1^, soil bulk density 1.5 g cm^-3^, 10.8g organic C kg^-1^, total N: 0.9 g kg^-1^, C:N ratio 12.2gkg^-1^, NH_4_^+^-N 2mg Kg^-1^, NO_3_ –N 29mg Kg^-1^ and 24 mg available Colwell P kg^-1^. The soil was sieved to 2 mm immediately after collection and stored at 4°C prior to potting in 2L undrained pots. We measured the above parameters by following standard methods (Rayment and Lyons, 2010).

The microbial inoculant and fertilizers were added separately to the soil according to the treatments (Table 1). The microbial inoculant powder was homogenously mixed into the top 3 cm soil. Fertilizers were also homogenously mixed into the top 3 cm soil in the respective treatments. The agricultural soil had been previously cropped with wheat and contained a community of naturally occurring AM fungi (*Rhizophagus irregularis)*. Wheat (*Triticum aestivum* L. cultivar: Wyalkatchem) was sown as it is widely grown in south-western Australia. Seeds were carefully selected for uniformity. Four seeds were sown in each pot, and the seedlings thinned to two per pot after emergence. The soil in each pot was watered and maintained at 70% (w/w basis) field capacity.

### Harvesting and Nutrient Analysis

Plant growth was assessed as plant height, number of tillers (Table 2), total biomass, grain quantity, 1000 grain weight, grain yield and nutrient concentration from shoot and grains. The number of generative tillers was counted just before flowering. Shoot and root fresh and dry weights were measured at tillering (7 weeks) and at maturity (12 weeks). Shoots were dried at 70°C in a forced-air oven for at least 72 h, weighed and finely ground. N was assessed using combustion analysis in an Elementar (vario Marco CNS; Elementar, Germany). Subsamples were digested in 5:0.5 HNO_3_-HClO_4_ (Simmons, 1975, 1978). P and K were measured using a Perkin Elmer Optima 5300dv instrument. Shoot N, P and K uptake were calculated as the product of shoot dry mass yield and the concentration of individual macro-element.

**Table 2:** Grain yield, harvest index, grain number per pot, and 1000 grain weight at maturity in the six treatments: control, microbial inoculant (Microbes), mineral fertilizer (MF), and three rates of chemical fertilizer [CF-1 (75 kg ha^-1^), CF-2 (55 kg ha^-1^) and CF-3 (43 kg ha^-1^)].

Treatment	Grain Yield (g pot^-1^)	Harvest index (%)	Grain number (pot^-1)^	1000 Grain weight (g)	Tiller number	
Control	3.80 ± 0.14a	86 ± 0.1c	81 ± 3.1a	47.13 ± 1.02a	4.0 ± 0a	
Microbes	4.41 ± 0.18b	85 ± 0.5bc	92 ± 6.3a	49.03 ± 0.93a	3.3 ± 0.14a	
MF	4.70 ± 0.16b	78 ± 0.2a	150 ± 2.5bc	47.09 ± 1.66a	6.5 ± 0c	
CF-1	4.66 ± 0.14b	76 ± 0.4a	169 ± 5.1c	46.62 ± 0.14a	6.4 ± 0.31c	
CF-2	4.41 ± 0.03ab	78 ± 0.9a	139 ± 8.4b	44.12 ± 0.35a	6.4 ± 0.24c	
CF-3	4.25 ± 0.07ab	83 ± 0.5b	95 ± 2.6a	44.79 ± 1.76a	5.3 ± 0.14b	
LSD_0.05_	0.404	1.6	15.3	3.84	0.539	
*P*-value	0.002	<0.001	<.001	0.147	<.001	


*Means followed by the same letter within a column are not significantly different according to LSD_*0.05*._*

The number of grains per ear was determined at maturity and 1000 grain weight was calculated (Table 2). Harvest index was calculated as the dry matter of grain yield divided by the sum of dry matter of grain yield and straw yield (Metho and Hammes, 2000). Wheat grains were ground prior to chemical analysis, and N, P and K uptake in grains were calculated as the product of grain dry mass yield and the concentration of individual macro-element.

### Soil DNA Extraction, PCR Amplification, and Sequencing

At each harvest, roots were carefully removed from the soil and shaken to remove loosely adhering soil. The more tightly adhering rhizosphere soil (Mickan et al., 2017) was collected and used for subsequent analysis of rhizosphere bacteria. Rhizosphere soil was transferred to a -20°C freezer prior to DNA extraction as described previously (Mickan et al., 2017). Paired end sequencing on the Illumina MiSeq was performed using the primer set of 27F-519R (Lane et al., 1985; Lane, 1991).

### Bioinformatics

Paired-end reads were assembled by aligning the forward and reverse reads using PEAR (version 0.9.5) (Zhang et al., 2014). Primers were identified, and trimmed sequences were processed using Quantitative Insights into Microbial Ecology (QIIME 1.8) (Caporaso et al., 2010) USEARCH (Edgar, 2010; Edgar et al., 2011) (version 8.0.1623) and UPARSE software. Using USEARCH tools sequences were quality filtered, and full-length duplicate sequences removed and sorted by abundance. Singletons or unique reads in the data set were discarded. Sequences were clustered following chimera filtering using the “RDP_Gold” database as a reference, Reads were mapped back to OTUs with a minimum identity of 97% to obtain the number of reads in each OUT. Using QIIME, taxonomy was assigned using the Greengenes database5 (Version 13_8, Aug 2013).

### Arbuscular Mycorrhizal (AM) Colonization in Roots

Roots were washed well with tap water to remove any remaining adhering soil particles, blotted dry with tissue paper, weighed, cut into 1 cm segments and mixed thoroughly. A 1g subsample of roots was taken and stained prior to assessment of AM fungal colonization (Abbott and Robson, 1981). After random dispersion of the stained roots in a 9-cm diameter Petri plate with gridlines, the percentage of root length colonized by AM fungi and meter of root length colonized were determined using the gridline intersect method (Giovannetti and Mosse, 1980) and estimated the root length (Newman, 1966).

### Soil Analysis at Harvest

EC and pH were measured (1:5 soil/water ratio) using a probe inserted into the water and 0.01M CaCl_2_, respectively. Soil pH was determined with a glass electrode (pH probe) using a soil-to-water ratio of 1:5 mixture and with CaCl_2_ as well. The soil EC was measured in water at 1:5 (w/v) ratios. Soil EC was also measured in CaCl_2_ at 1:5 (w/v) ratios.

### Statistical Analysis

One-way analysis of variance (ANOVA) was carried out using Genstat 18th edition (64 bit) software for wheat growth, mycorrhizal colonization, basic soil data and wheat nutrient data for shoots and grain. Main effects were compared using the least significant difference (LSD) for multiple comparison tests, where *P* ≤ 0.05 was considered the threshold value for significance. The effect of treatments on wheat biomass, grain yield and components of yield were investigated using ANOVA.

To assess bacterial community assemblages and alpha (α) diversity, a two-way ANOVA was applied to test for the effects of ‘Fertilizer’ and ‘Harvest time’ treatments on bacterial relative abundance and alpha diversity. To assess the OTU level community data, a non-metric multidimensional scaling (NMDS) plot of soil bacterial community at tillering and maturity, and fertilizer treatments using Bray-Curtis dissimilarity matrix method (97% similarity). A permutational multivariate analysis of variance (PERMANOVA) was used to test the significant difference between taxonomic bacterial (OTU level) data (beta diversity) and treatments (fertilizer and harvest time) using 999 permutations in the ‘vegan’ package (Oksanen et al., 2013).

## Results

### Plant Growth and Yield

**FIGURE 1:**
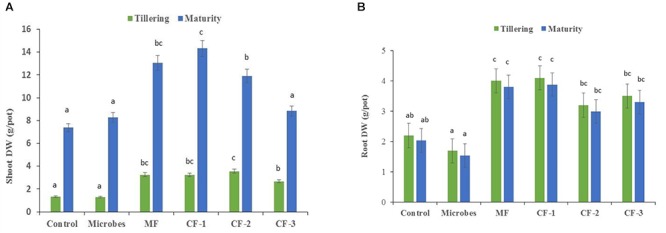
**(A)** Shoot dry weight and **(B)** root dry weight at tillering and maturity in the six treatments: control, microbial inoculant (Microbes), mineral fertilizer (MF), and three rates of chemical fertilizer [CF-1 (75 kg ha^-1^), CF-2 (55 kg ha^-1^), and CF-3 (43 kg ha^-1^)]. Mean data followed by a similar letter(s) are not statistically significant within each sampling time.

There were significant differences between the microbial inoculant, mineral and chemical fertilizer treatments (*P*<0.001), with the greatest effects on shoot and root biomass in the fertilizer treatments relative to the microbial inoculant and the untreated control at tillering and maturity (Figures 1A,B).

Shoot and root dry weights were unaffected by the application of the microbial inoculant (*P* < 0.05; Figures 1A,B). At tillering, shoot dry weight increased with the application of mineral and chemical fertilizer (Figure 1A). Total shoot dry weight accumulation increased with increasing levels of chemical fertilizer, especially at maturity (Figure 1A). The lower chemical fertilizer rate had little effect on plant growth, while the higher fertilizer rate increased shoot growth (Figure 1A). A similar trend was observed for root dry weight, except with the CF-2 (55 kg ha^-1^) treatment (Figure 1B).

The microbial inoculant produced less shoot and root dry weight at tillering and maturity than did the mineral and chemical fertilizers when applied at equivalent rates (Figures 1A,B). At maturity, the microbial inoculant produced similar shoot dry weight to the lowest rate of chemical fertilizer (43 kg ha^-1^), but less than the two higher rates (55 and 75 kg ha^-1^), and lower root dry weight than the three chemical fertilizer treatments (Figure 1B).

The microbial inoculant increased grain yield relative to the control (*P* < 0.05, Table 2). Harvest index was not affected by the microbial inoculant (*P* > 0.05, Table 2) but was decreased with all fertilizer treatments relative to the control. Thousand grain weight was not affected by any of the treatments (*P* > 0.05, Table 2). The mineral fertilizer and chemical fertilizer treatments generally increased grain number (Table 2); the microbial inoculant produced the higher grain yield than the control, but it did not differ from any of the fertilizer treatments (Table 2).

### Nutrient Concentration and Uptake

The microbial inoculant and mineral fertilizer treatments had no effect on shoot N concentration at tillering and maturity, except for an increase with 75 kg ha^-1^ chemical fertilizer at tillering but not at maturity (Table 3). The shoot P concentration of wheat treated with the microbial inoculant was similar to that of the control at tillering but was higher with the mineral and chemical fertilizer treatments. At maturity, there was no difference in P concentration of shoots treated with the microbial inoculant and chemical fertilizer compared with the control, but it was lower for the mineral fertilizer (Table 3). Wheat treated with the microbial inoculant had a similar shoot K concentration to that of all other treatments at tillering, but at maturity, it was lower than the two higher rates of chemical fertilizer (55 and 75 kg ha^-1^).

**Table 3:** Shoot N, P, and K concentrations at tillering and maturity in the six treatments: control, microbial inoculant (Microbes), mineral fertilizer (MF), and three rates of chemical fertilizer [CF-1 (75 kg ha^-1^), CF-2 (55 kg ha^-1^), and CF-3 (43 kg ha^-1^)].

Treatment	Tillering			Maturity		
	*N* (%)	*P* (%)	*K* (%)	*N* (%)	*P* (%)	*K* (%)
Control	2.19 ± 0.02a	0.36 ± 0.02ab	3.79 ± 07ab	0.35 ± 0.01a	0.31 ± 0.01b	1.9 ± 0.09ab
Microbes	2.40 ± 0.08a	0.34 ± 0.03a	3.95 ± 0.09ab	0.35 ± 0.04a	0.25 ± 0.03ab	1.6 ± 0.17a
MF	2.58 ± 0.17a	0.46 ± 0.02b	4.00 ± 0.19b	0.40 ± 0.02a	0.17 ± 0.01a	2.1 ± 0.09ab
CF-1	3.15 ± 0.16b	0.62 ± 0.02c	4.38 ± 0.21b	0.40 ± 0.02a	0.30 ± 0.02b	2.4 ± 0.12b
CF-2	2.57 ± 0.10a	0.61 ± 0.02c	4.13 ± 0.17b	0.37 ± 0.02a	0.26 ± 0.02b	2.3 ± 0.13b
CF-3	2.33 ± 0.08a	0.58 ± 0.01c	3.31 ± 0.06a	0.35 ± 0.03a	0.28 ± 0.02b	1.9 ± 0.09ab
LSD_0.05_	0.354	0.078	0.44	0.077	0.056	0.356
*P-*value	<0.001	<0.001	0.002	0.472	<0.001	0.002


*Means followed by the same letter within a column are not significantly different according to LSD _*0.05*._*

The N, P, and K uptake by shoots were significantly influenced by treatments both at tillering and maturity (Supplementary Table S1; *p*<0.001). At both tillering and maturity, wheat inoculated with the microbial inoculant had similar N, P, and K uptake to the control plants, but lower than the fertilized plants, except for P and K uptake which was similar to 43 kg/ha chemical fertilizer (Supplementary Table S1).

Wheat inoculated with the multiple species microbial inoculant had similar grain N concentration compared to all other treatments, but it was reduced with all fertilizer treatments compared to control (Supplementary Table S2). Grain P concentration was higher with the mineral and chemical fertilizer at 75 kg ha^-1^, but there was no effect of the microbial inoculant. Grain K concentration was the same for the microbial inoculant and the control but lower with the fertilizer treatments (Supplementary Table S2).

Application of the microbial inoculant did not increase grain N uptake. P uptake in grain with the microbial inoculant was same as that of the control and chemical fertilizer applied at both 43 and 55 kg ha^-1^ (Supplementary Table S2). Microbial inoculant application increased K uptake in wheat grain compared to all fertilizer treatments (Supplementary Table S2).

### Soil pH

Although the overall variation in soil pH was moderate, ranging from 4.5 to 5.8, there was a significant difference in pH between soils receiving the microbial inoculant and mineral fertilizer treatments (*P* = 0.05, Supplementary Table S3). Application of the microbial inoculant significantly increased soil pH (measured in water and CaCl_2_) at maturity compared to all other fertilizer treatments.

### AM Fungal Colonization

**FIGURE 2:**
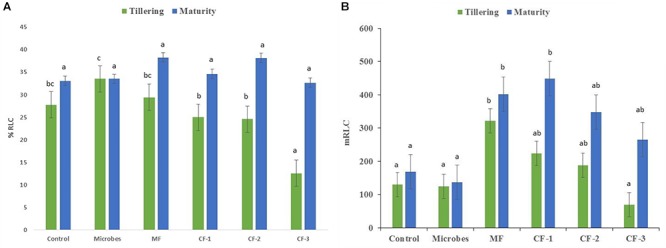
**(A)** The percentage of root length colonized by mycorrhiza (% RLC) and **(B)** root length colonized by mycorrhiza (m RLC) at tillering and maturity in the six treatments: control, microbial inoculant (Microbes), mineral fertilizer (MF), and three rates of chemical fertilizer [CF-1 (75 kg ha^-1^), CF-2 (55 kg ha^-1^), and CF-3 (43 kg ha^-1^)]. Mean data followed by a similar letter(s) are not statistically significant within each sampling time.

Mycorrhizal colonization was recorded at both tillering and maturity (Figure 2A). Colonization (%) increased with application of the microbial inoculant compared to all chemical fertilizer treatments at tillering but not at maturity (Figure 2A). The mineral fertilizer treatments had the highest length of root colonized by AM fungi at tillering relative to all other treatments except the chemical fertilizer at 55 and 75 kg ha^-1^, and was greatest at maturity for chemical fertilizer applied at 75 kg ha^-1^ (Figure 2B) compared to the microbial inoculant.

### Soil Bacterial Community Composition and Diversity

**FIGURE 3:**
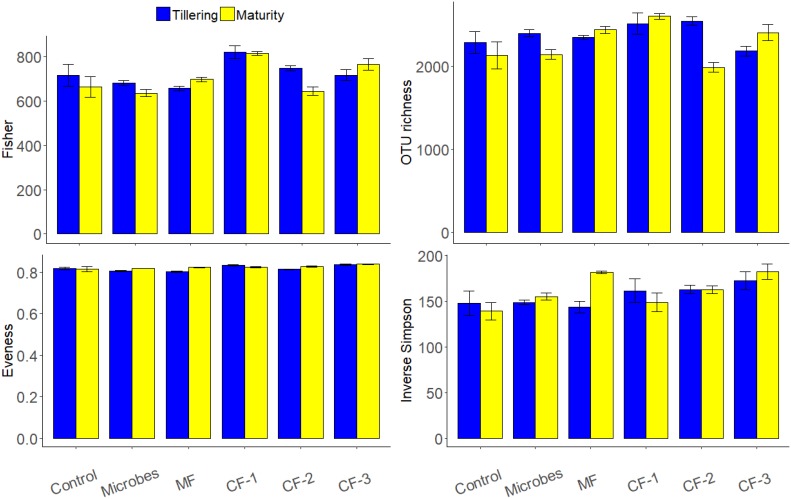
Alpha diversity indices based on OTU composition (97% similarity) on the effect of ‘fertilizer treatments,’ ‘harvests’ (tillering and maturity), and their interaction for the six treatments: control, microbial inoculant (Microbes), mineral fertilizer (MF), and three rates of chemical fertilizer [CF-1 (75 kg ha^-1^), CF-2 (55 kg ha^-1^), and CF-3 (43 kg ha^-1^)].

The bacterial phyla *Proteobacteria* (36%) and *Actinobacteria* (35%) were dominant in all rhizosphere soil for treatments (Figure 3). Alpha diversity showed that the fertilizer treatments altered Evenness (*P* < 0.001) and OTU richness (*P* = 0.03) as well as the diversity index of Inverse Simpson (*P* = 0.005) and Fisher (*P* < 0.001) (Supplementary Table S4). There were no significant effects of treatments at maturity. However, there were interactions between fertilizer and harvesting time for OTU richness (*P* < 0.001) and Fisher (*P* = 0.042).

**FIGURE 4:**
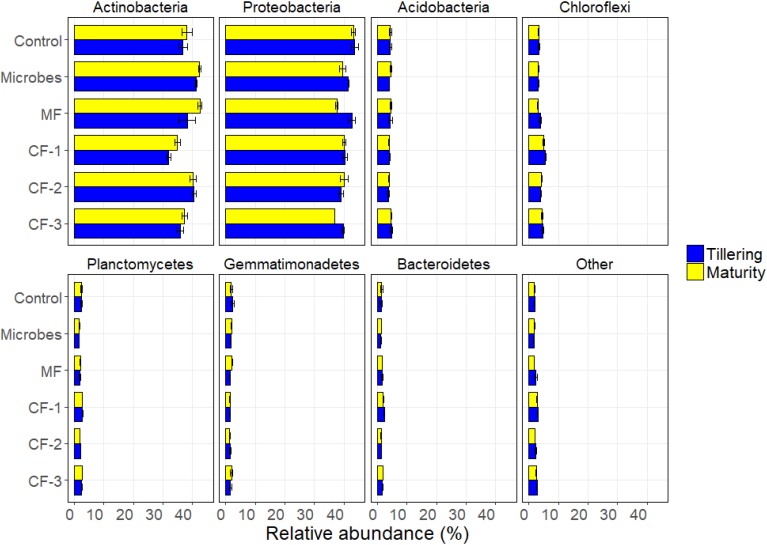
Relative abundance of soil bacteria at phylum resolution in the six treatments: control, microbial inoculant (Microbes), mineral fertilizer (MF), and three rates of chemical fertilizer [CF-1 (75 kg ha^-1^), CF-2 (55 kg ha^-1^), and CF-3 (43 kg ha^-1^)].

The relative abundance at phylum level varied with the fertilizer treatments for *Actinobacteria* (*P* < 0.001), *Proteobacteria* (*P* < 0.001), *Chloroflexi* (*P* < 0.001), *Planctomycetes* (*P* < 0.001), *Firmicutes* (*P* < 0.001), and *Bacteroidete*s (*P* < 0.001). The relative abundance of *Actinobacteria* (*P* = 0.016), *Proteobacteri*a (*P* = 0.001), and *Firmicutes* (*P* < 0.001) varied with harvesting time. There were interactions between fertilizer and harvest time for *Firmicutes* (*P* < 0.001) and for *TM7* (*P* = 0.001) (Supplementary Table S5 and Figure 4).

### Impact of Fertilizer Treatments and Harvesting Time on Bacterial OTU Community Composition

**FIGURE 5:**
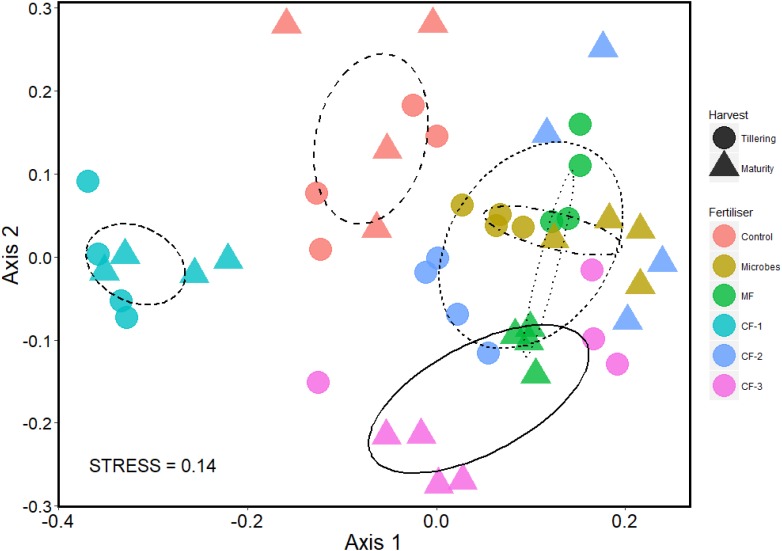
Non-metric multidimensional scaling (NMDS) plot of OTU community assemblage analysis based on 97% similarity OTU abundance data (square root transformed), using 999 permutations in the six treatments: control, microbial inoculant (Microbes), mineral fertilizer (MF), and three rates of chemical fertilizer[CF-1 (75 kg ha^-1^), CF-2 (55 kg ha^-1^), and CF-3 (43 kg ha^-1^)].

A NMDS plot was used to visualize the community assemblages at the OTU level. There was distinct clustering of all treatments, although the microbial inoculant and the mineral fertilizer treatments had similar community assemblages (Figure 5). PERMANOVA revealed that the fertilizer treatments significantly altered the community composition at the OTU level (*P* < 0.001), and it also changed with time of harvest (*P* = 0.003). There was an interaction between fertilizer and harvest time (*P* < 0.001) (Supplementary Table S6).

## Discussion

We hypothesized that a mixed microbial inoculant would be as effective as a mineral fertilizer applied at the recommended level for wheat growth and yield in the agricultural soil used. Indeed, the microbial inoculant significantly increased grain yield without increasing shoot growth of wheat.

The wheat response to N and P fertilizer (a conventional chemical fertilizer) and the low soil organic matter content in the soil indicated that the mineralization potential of this soil was far below the N and P requirements of the wheat crop. Previous studies with different microbial inoculants have shown increased plant height and spikelets per spike in various crops (e.g., Khalid et al., 1997; Biswas et al., 2000b). Several studies reported increased seed P content associated with the application of phosphate-solubilising microorganisms (Kucey, 1987; Mehana and Wahid, 2002; Zaidi et al., 2004). However, the microbial inoculant used in our study did not affect P concentration but did increase N and K concentrations (*P* < 0.001).

The mineral fertilizer was expected to be less effective than the chemical fertilizer when applied at equivalent levels of N and P because of differences in solubility of these nutrients. Unexpectedly, for grain yield, the mineral fertilizer was as effective as all of the chemical fertilizer treatments. For shoot growth, the mineral fertilizer was as effective as the chemical fertilizer when applied at equivalent levels of N and P at tillering, and when applied at equivalent levels of N at maturity.

It was expected that the mechanisms underlying potential benefits of the microbial inoculant in this study would be linked to soil bacterial diversity in the rhizosphere and root colonization by AM fungi. The microbial inoculant was expected to complement the existing bacterial community in the rhizosphere, but relatively minor changes in the bacterial community composition were recorded at maturity. In relation to mycorrhizal colonization, it was expected that the microbial inoculant and mineral fertilizer would not influence colonization of the wheat roots by AM fungi, but the chemical fertilizer would reduce the extent by which roots were colonized by AM fungi. However, mycorrhizal colonization of roots increased with application of the microbial inoculant at an early stage of plant growth (tillering) where benefits to the plant could be expected. In contrast, as expected, the chemical fertilizer decreased mycorrhizal colonization, which could reduce potential benefits compared to plants with higher AM fungal colonization in the microbial inoculant treatment. Low nutrient availability stresses plants, which leads to the development of longer and more active roots with mycorrhizas that scavenge for nutrients (Hui-Lian, 2001).

The microbial inoculant used in our study contained a group of beneficial microorganisms that promoted plant growth and increased grain yield in the absence of chemical fertilizer. Based on previous studies, this could be attributed to increased photosynthetic capacity and nutrient availability (Hui-Lian, 2001). Overall, the application of fertilizer to this soil had little effect on the bacterial community composition, with increases in relative abundance of only two of the top 25 most-abundant OTUs (*Proteobacteria* and *Actinobacteria*). Farmers have anecdotally reported increases in plant growth, yield and grain quality following application of a range of microbial inoculants. Further studies are needed to examine the mechanisms of how microbial inoculants influence plant growth to elucidate issues confronted in the technology of microbial inoculant development.

## Conclusion

The multiple species microbial inoculant stimulated grain yield of wheat to the same level as did the fertilizers applied to the moderately N and P deficient soil used in this experiment. While microbial inoculation is unlikely to significantly change the abundance and composition of indigenous microbial communities, the localized intervention via inoculation has the potential to contribute to nutrient cycling in the wheat rhizosphere. Combinations of microbial inoculants and fertilizers of different elemental solubility should be investigated to identify alternative strategies for increasing profitability and sustainability of crop production, with greater emphasis placed on the role of beneficial soil microorganisms.

## Author Contributions

SA conducted the experiment, analyzed the data, and wrote the manuscript. LA, KS, and ZS supervised the work, helped to develop the experiments, revised the manuscript, and contributed to the writing. AW has revised the manuscript and contributed to the writing. BM helped in bioinformatics and interpretation of results.

## Conflict of Interest Statement

The authors declare that the research was conducted in the absence of any commercial or financial relationships that could be construed as a potential conflict of interest.

## References

[B1] Abbott K. L. Robson A. (1981). Infectivity and effectiveness of vesicular arbuscular mycorrhizal fungi. *Aust. J. Agric. Res.* 32 631–639. 10.1071/AR9810631

[B2] Adesemoye A. Q. Torbert H. A. Kloepper J. W. (2008). Enhanced plant nutrient use efficiency with PGPR and AMF in an integrated nutrient management system. *Can. J. Microbiol.* 54 876–886. 10.1139/W08-081 18923557

[B3] Ahemad M. Kibret M. (2014). Mechanisms and applications of plant growth promoting rhizobacteria: current perspective. *J. King Saud Uni – Sci.* 26 1–20. 10.1016/j.jksus.2013.05.001

[B4] Bakken A. K. Gautneb H. Sveistrup T. Myhr K. (2000). Crushed rocks and mine tailings applied as K fertilisers on grassland. *Nutr. Cycl. Agroecosys.* 56 53–57. 10.1023/A:1009709914578

[B5] Barker W. W. Welch S. A. Banfield J. F. (1997). Biogeochemical weathering of silicate minerals. *Rev. Mineral. Geochem.* 35 391–428.

[B6] Barrow N. J. Malajczuk N. Shaw T. C. (1977). A direct test of the ability of vesicular-arbuscular mycorrhiza to help plants take up fixed soil phosphate. *New Phytol.* 78 269–276. 10.1111/j.1469-8137.1977.tb04830.x

[B7] Biswas J. C. Ladha J. K. Dazzo F. B. (2000a). Rhizobia inoculation improves nutrient uptake and growth of lowland rice. *Soil Sci. Soc. Am. J.* 64 1644–1650. 10.2136/sssaj2000.6451644x

[B8] Biswas J. C. Ladha J. K. Dazzo F. B. Yanni Y. G. Rolfe B. G. (2000b). Rhizobial inoculation influences seedling vigor and yield of rice. *Agron. J.* 92 880–886. 10.2134/agronj2000.925880x

[B9] Brown P. Saa S. (2015). Biostimulants in agriculture. *Front. Plant Sci.* 6:671. 10.3389/fpls.2015.00671 26379695 PMC4550782

[B10] Calvo V. P. Nelson L. Kloepper J. (2014). Agricultural uses of plant biostimulants. *Plant Soil* 383 3–41. 10.1007/s11104-014-2131-8

[B11] Caporaso J. G. Kuczynski J. Stombaugh J. Bittinger K. Bushman F. D. Costello E. K. (2010). QIIME allows analysis of high-throughput community sequencing data. *Nat. Methods* 7 335–336. 10.1038/nmeth.f.303 20383131 PMC3156573

[B12] Carson J. Harley A. Abbott L. K. Gleeson (2012). *Adding Value to Organic Pasture: Microbes and Minerals.* Kingston, ACT: Rural Industries Research and Development Corporation.

[B13] Coroneos C. Hinsinger P. Gilkes R. J. (1996). Granite powder as a source of potassium for plants: a glasshouse bioassay comparing two pasture species. *Fert. Res.* 45 143–152. 10.1007/BF00790664

[B14] Deubel A. Merbach W. (2005). “Influence of microorganisms on phosphorus bioavailability in soils,” in *Microorganisms in Soils: Roles in Genesis and Functions* eds Buscot F. Varma A. (Berlin: Springer-Verlag) 62.

[B15] Dutton V. M. Evans C. S. (1996). Oxalate production by fungi: its role in pathogenicity and ecology in the soil environment. *Can. J. Microbiol.* 42 881–895. 10.1139/m96-114

[B16] Edgar R. C. (2010). Search and clustering orders of magnitude faster than BLAST. *Bioinformatics* 26 2460–2461. 10.1093/bioinformatics/btq461 20709691

[B17] Edgar R. C. Haas B. J. Clemente J. C. Quince C. Knight R. (2011). UCHIME improves sensitivity and speed of chimera detection. *Bioinformatics* 27 2194–2200. 10.1093/bioinformatics/btr381 21700674 PMC3150044

[B18] Fankem H. Nwaga D. Deubel A. Dieng L. Merbach W. Etoa F. X. (2006). Occurrence and functioning of phosphate solubilizing microorganisms from oil palm tree (*Elaeis guineensis*) rhizosphere in Cameroon. *Afr. J. Biotech.* 5 2450–2460.

[B19] Fleming C. Sharma S. H. Selby C. H. Rao J. R. Trevor M. (2014). Plant biostimulantsl: a review on the processing of macroalgae and use of extracts for crop management to reduce abiotic and biotic stresses. *J. Appl. Phycol.* 26 465–490. 10.1007/s10811-013-0101-9

[B20] Gillmann G. P. Burkett D. C. Coventry R. J. (2002). Amending highly weathered soils with finely ground basalt rock. *Appl. Geochem.* 17 987–1001. 10.1016/S0883-2927(02)00078-1

[B21] Giovannetti M. Mosse B. (1980). An evaluation of techniques for measuring vesicular mycorrhizal colonization in roots. *New Phytol.* 84 489–500. 10.1111/j.1469-8137.1980.tb04556.x

[B22] Goldstein A. H. (1996). “Involvement of the quinoprotein glucose dehydrogenase in the solubilisation of exogenous phosphates by Gram-negative bacteria,” in *Phosphate in Microorganisms: Cellular and Molecular Biology* eds Torriani-Gorini A. Yagi E. Silver S. (Washington, DC: ASM Press) 197–203.

[B23] He Z. L. Zhu J. (1988). Microbial utilization and transformation of phosphate adsorbed by variable charged minerals. *Soil Biol. Biochem.* 30 917–923. 10.1016/S0038-0717(97)00188-0

[B24] Hinsinger P. Barros O. N. F. Beneditti M. F. Noack Y. Callot G. (2001). Plant-induced weathering of a basaltic rock: experimental evidence. *Geochim. Cosmochim. Acta* 65 137–152. 10.1016/S0016-7037(00)00524-X

[B25] Hinsinger P. Bolland M. D. A. Gilkes R. J. (1996). Silicate rock powder: effect on selected chemical properties of a range of soils from Western Australia and on plant growth as assessed in a glasshouse experiment. *Fert. Res.* 45 69–79. 10.1007/BF00749883

[B26] Hui-Lian X. (2001). Effects of a microbial inoculant and organic fertilizers on the growth, photosynthesis and yield of sweet corn. *J. Crop. Prod.* 3 183–214. 10.1300/J144v03n01_16

[B27] Khalid A. Arshad M. Zahir Z. A. Khaliq A. (1997). potential of plant growth promoting rhizobacteria for enhancing wheat yield. *J. Anim. Plant Sci.* 7 53–56.

[B28] Kucey R. M. (1987). Increased phosphorus uptake by wheat and field beans inoculated with a phosphorus-solubilizing *Penicillium bilaji* Strain and with vesicular-arbuscular mycorrhizal fungi. *Appl. Environ. Microbiol.* 53 2699–2703. 16347487 10.1128/aem.53.12.2699-2703.1987 PMC204184

[B29] Lane D. J. (1991). “Nucleic acid techniques in bacterial systematics,” in *16S/23S rRNA Sequencing* eds Stackebrandt E. Goodfellow M. (New York, NY: Wiley) 115–175.

[B30] Lane D. J. Pace B. Olsen G. J. Stahl D. A. Sogin M. L. Pace N. R. (1985). Rapid determination of 16S ribosomal RNA sequences for phylogenetic analyses. *Proc. Natl. Acad. Sci. U.S.A.* 82 6955–6959. 10.1073/pnas.82.20.6955 2413450 PMC391288

[B31] Loganathan P. Hedley M. J. Bolan N. S. Currie L. D. (2002). Field evaluation of the liming value of two phosphate rocks and their partially acidulated products after 16 years of annual application to grazed pasture. *Nutr. Cycle Agroecosys.* 72 287–297. 10.1007/s10705-005-4277-5

[B32] Mehana T. A. Wahid O. A. A. (2002). Associative effect of phosphate dissolving fungi, *Rhizobium* and phosphate fertilizer on some soil properties, yield components and the phosphorus and nitrogen concentration and uptake by *Vicia faba* L. under field conditions. *Pak. J. Biol. Sci.* 5 1226–1231. 10.3923/pjbs.2002.1226.1231

[B33] Metho L. A. Hammes P. S. (2000). The harvest index of individual ears of four South African wheat (*Triticum aestivum* L.) cultivars. *South Afr. J. Plant Soil* 17 144–146. 10.1080/02571862.2000.10634887

[B34] Mickan B. Hart M. M. Solaiman Z. M. Jenkins S. N. Siddique K. H. M. Abbott L. K. (2017). Molecular divergence of fungal communities in soil, roots and hyphae highlight the importance of sampling strategies. *Rhizosphere* 4 104–111. 10.1016/j.rhisph.2017.09.003

[B35] Nahas E. (1996). Factors determining rock phosphate solubilization by microorganism isolated from soil. *World J. Microb. Biotechnol.* 12 18–23. 10.1007/BF00327716 24415416

[B36] Newman E. I. (1966). A method of estimating the total length of a root in a smaple. *J. Appl. Ecol.* 3 139–145. 10.2307/2401670

[B37] Oksanen J. Blanchet F. G. Kindt R. Legendre P. Minchin P. R. O’Hara R. B. (2013). *Vegan: Community Ecology Package.* Available at: https://cran.r-project.org/web/packages/vegan/index.html

[B38] Pairunan A. K. Robson A. D. Abbott L. K. (1980). The effectiveness of vesicular-arbuscular mycorrhizas in increasing growth and phosphorus uptake of subterranean clover from phosphorus sources of different solubilities. *New Phytol.* 84 327–338. 10.1111/j.1469-8137.1980.tb04433.x

[B39] Priyono J. Gilkes R. (2004). Dissolution of milled-silicate rock fertilisers in the soil. *Aus. J. Soil Res.* 42 441–448. 10.1071/SR03138

[B40] Rayment G. E. Lyons D. J. (2010). *Soil Chemical Methods.* Melbourne, VIC: Australasia CSIRO Publishing

[B41] Richardson A. E. (1994). *Soil Microorganisms and Phosphorus Availability in Soil Biota: Management in Sustainable Farming Systems,* eds Pankhurst C. E. Melbourne, VIC: CSIRO, 50–62.

[B42] Richardson A. E. (2001). Prospects for using soil microorganisms to improve the acquisition of phosphorus by plants. *Aus. J. Plant. Physiol.* 28 897–906.

[B43] Rodriguez H. Fraga R. (1999). Phosphate solubilizing bacteria and their role in plant growth promotion. *Biotechnol. Adv.* 17 319–339. 10.1016/S0734-9750(99)00014-2 14538133

[B44] Rodriguez H. Gonzalez T. Bashan Y. (2006). Genetics of phosphate solubilization and its potential applications for improving plant growth – Promoting bacteria. *Plant Soil* 287 15–21. 10.1007/s11104-006-9056-9

[B45] Roger J. R. Bennett P. C. (2004). Mineral stimulation of subsurface microorganisms: release of limiting nutrients from silicates. *Chem. Geol.* 203 91–108. 10.1016/j.chemgeo.2003.09.001

[B46] Saa S. Rio A. O. Castro S. Brown P. H. (2015). Foliar application of microbial and plant based biostimulants increases growth and potassium uptake in almond (*Prunus dulcis* [Mill.] D.A,Webb). *Front. Plant. Sci.* 87:6. 10.3389/fpls.2015.00087 25755660 PMC4337363

[B47] Sanz S. J. I. Rowell D. L. (1988). The use of feldspars as potassium fertilizers in the savannah of Colombia. *Fert. Res.* 17 71–83. 10.1007/BF01050458

[B48] Schmitz O. J. Buchkowski R. W. Burghardt K. T. Donihue C. M. (2015). Functional traits and trait-mediated interactions: connecting community-level interactions with ecosystem functioning. *Adv. Ecol. Res.* 52 319–343. 10.1016/bs.aecr.2015.01.003

[B49] Simmons W. J. (1975). Background absorption error in determination of copper in plants by flame atomic absorption spectrometry. *Anal. Chem.* 50 870–873. 10.1021/ac50029a014

[B50] Simmons W. J. (1978). Determination of low concentrations of cobalt in small samples of plant material by flameless atomic absorption spectrophotometry. *Anal. Chem.* 47 2015–2018. 10.1021/ac60362a013 4756749

[B51] Smith S. E. Smith F. A. (2012). Fresh perspectives on the roles of arbuscular mycorrhizal fungi in plant nutrition and growth. *Mycologia* 104 1–13. 10.3852/11-229 21933929

[B52] Smith S. E. Read D. J. (eds). (2008) *Mycorrhizal Symbiosis* 3rd Edn. Cambridge: Academic press.

[B53] Surange S. Wollum A. G. Kumar N. Nautiyal C. S. (1995). Characterization of *Rhizobium* from root nodules of leguminous trees growing in alkaline soils. *Can. J. Microbiol.* 43 891–894. 10.1139/m97-130

[B54] Zaidi A. Khan M. S. Aamil M. (2004). Bioassociative effect of rhizospheric microorganisms on growth, yield, and nutrient uptake of greengram. *J. Plant Nutr.* 27 601–612. 10.1081/PLN-120030370

[B55] Zhang J. Kobert K. Flouri T. Stamatakis A. (2014). PEAR: a fast and accurate illumina paired-end reAd mergeR. *Bioinformatics* 30 614–620. 10.1093/bioinformatics/btt593 24142950 PMC3933873

