# Supplementary material for: Response of Wheat to a Multiple Species Microbial Inoculant Compared to Fertilizer Application
Source: Front Plant Sci. 2018 Nov 13;9:1601. doi: 10.3389/fpls.2018.01601 (PMC6243077; doi:10.3389/fpls.2018.01601)
Supplement: Supplementary file 1 [file Table_1.pdf]

Response of wheat to a multiple species microbial inoculant compared to fertiliser application

Salmabi K. Assainar[1,2*], Lynette K. Abbott[1,2], Bede S. Mickan[1,2,3], Andrew S. Whiteley[1,2], Kadambot H. M. Siddique[1,2], and Zakaria M. Solaiman[1,2]

TABLE S1. Shoot N, P, and K uptake at tillering and maturity in the six treatments: control, microbial inoculant (Microbes), mineral fertiliser (MF), and three rates of chemical fertiliser [CF-1 (75 kg ha^{-1}), CF-2 (55 kg ha^{-1}) and CF-3 (43 kg ha^{-1})].

Treatment	Tillering			Maturity		
	N uptake (mg pot^{-1})	P uptake (mg pot^{-1})	K uptake (mg pot^{-1})	N uptake (mg pot^{-1})	P uptake (mg pot^{-1})	K uptake (mg pot^{-1})
Control	29.6±1.0a	4.9±0.4a	51.1±1.5a	25.9±0.9a	22.7±0.3ab	138.9±5.4a
Microbes	30.7±0.6a	4.3±0.3a	50.6±0.9a	29.2±2.8a	20.5±2.6a	135.6±17.5a
MF	83.2±2.8c	15.0±1.0b	129.5±3.0c	52.8±3.4bc	21.5±0.7a	275.5±21.8b
CF-1	101.5±3.9d	20.2±1.5cd	142.2±10.9c	57.4±1.9c	43.0±3.1c	345.7±11.9c
CF-2	91.1±3.9cd	21.6±0.5d	146.2±2.8c	44.3±0.4b	31.0±1.0b	274.2±15.5b
CF-3	62.0±5.4b	15.7±1.5bc	88.0±6.3b	30.8±2.9a	25.2±2.0ab	171.4±9.3a
LSD(≤0.05)	10.04	3.01	16.2	7.0	5.7	43.4
P value	<.001	<.001	<0.001	<.001	<.001	<.001

Means followed by the same letter within a column are not significantly different according to $LSD_{0.05}$.

TABLE S2. Grain N, P and K concentrations and uptake at maturity in the six treatments: control, microbial inoculant (Microbes), mineral fertiliser (MF), and three rates of chemical fertiliser [CF-1 (75 kg ha^{-1}), CF-2 (55 kg ha^{-1}) and CF-3 (43 kg ha^{-1})].

Treatment	N (%)	P (%)	K (%)	N uptake (mg pot^{-1})	P uptake (mg pot^{-1})	K uptake (mg pot^{-1})
Control	2.1±0.02b	0.018±0.001ab	7.0±0.24c	79.6±3.1a	0.7±0.1a	263.7±7.1bc
Microbes	2.0±0.09ab	0.017±0.000a	6.5±0.37bc	86.8±3.5a	0.7±0.1a	284.3±13.3c
MF	1.8±0.06a	0.022±0.000bc	4.2±0.30a	83.7±5.3a	1.0±0.1bc	194.9±8.5a
CF-1	1.8±0.02a	0.024±0.001c	4.0±0.26a	83.3±3.5a	1.1±0.1c	188.1±13.9a
CF-2	1.8±0.09a	0.019±0.001abc	4.2±0.28a	80.0±4.9a	0.9±0.1ab	186.8±13.1a
CF-3	1.7±0.04a	0.020±0.000abc	5.2±0.13ab	73.8±1.4a	0.9±0.1ab	222.6±8.1ab
$LSD_{0.05}$	0.186	0.003	0.82	11.4	0.1	32.7
P value	0.005	<0.001	<0.001	0.276	<0.001	<0.001

Means followed by the same letter within a column are not significantly different according to $LSD_{0.05}$.

TABLE S3. Soil pH (water), pH ($CaCl_2$) and EC at tillering and maturity in the six treatments: control, microbial inoculant (Microbes), mineral fertiliser (MF), and three rates of chemical fertiliser [CF-1 (75 kg ha^{-1}), CF-2 (55 kg ha^{-1}) and CF-3 (43 kg ha^{-1})].

Treatment	Tillering			Maturity		
	EC (μS cm^{-1})	pH (water)	pH ($CaCl_2$)	EC (μS cm^{-1})	pH (water)	pH ($CaCl_2$)
Control	83.4±16.2a	5.29±0.09c	5.09±0.06d	36.6±1.2a	5.83±0.03c	5.4 ±0.02c
Microbes	82.3±12.1a	5.32±0.06c	5.15±0.03d	45.4±1.7a	5.83±0.05c	5.5±0.01c
MF	102.3±3.5a	4.98±0.04bc	4.87±0.04cd	85.5±5.3b	5.29±0.03b	5.1±0.06b
CF-1	108.3±4.0a	4.55±0.12a	4.36±0.07a	81.3±6.6 b	5.01±0.10a	4.8±0.14a
CF-2	87.8±4.1a	4.77±0.09ab	4.76±0.12bc	73.0±3.8b	4.92±0.05a	4.8±0.02a
CF-3	70.5±1.5a	4.57±0.07a	4.50±0.06ab	71.5±5.3b	4.82±0.03a	4.7±0.02a
$LSD_{0.05}$	25.8	0.26	0.212	13.23	0.169	0.188
P value	0.065	<0.001	<0.001	<0.001	<0.001	<0.001

Means followed by the same letter within a column are not significantly different according to $LSD_{0.05}$.

TABLE S4. Two-way ANOVA of alpha diversity indices based on OTU composition (97% similarity) on the effect of different 'fertiliser treatments' and 'harvesting time' (first and second), and the interaction between 'fertiliser treatments' and 'harvesting time'.

		Degrees of freedom	Sum of	Mean Squares	F	Pr(>F)
Evenness	Fertiliser	5	5390.1	1078.01	3.94	<0.001
	Harvest	1	369.4	369.37	1.35	0.252
	Fertiliser ×Harvest	5	3314.1	662.82	2.42	0.054
	Residuals	36	9838.5	273.29		
OTU richness	Fertiliser	5	637375	127475	4.30	0.003
	Harvest	1	111265	111265	3.75	0.060
	Fertiliser ×Harvest	5	828866	165773	5.60	<0.001
	Residuals	36	1065590	29600		
Inverse Simpson	Fertiliser	5	5390.1	1078.01	3.94	0.005
	Harvest	1	369.4	369.37	1.35	0.252
	Fertiliser ×Harvest	5	3314.1	662.82	2.42	0.054
	Residuals	36	9838.5	273.29		
Fisher	Fertiliser	5	134645	26929	10.23	<0.001
	Harvest	1	4812	4812.4	1.82	0.184
	Fertiliser ×Harvest	5	33980	6796.1	2.58	0.042
	Residuals	36	94727	2631.3		

TABLE S5. Two-way ANOVA results showing *P* values fixed at Phylum resolution of relative abundance. Treatments were: control, microbial inoculant (microbes), mineral fertiliser (MF), and chemical fertiliser (CF) in different concentrations like75kg ha^{-1} (CF-1), 55 kg ha^{-1} (CF-2) and 43kg ha-1 (CF-3). Significant *P* values indicated by * and *** corresponding to *P* < 0.05 and < 0.001, respectively.

	Fertiliser		Harvest time		Fertiliser x Harvest time	
	F	P	F	P	F	P
Actinobacteria	12.59	<0.001***	6.32	0.016*	0.86	0.515
Proteobacteria	8.90	<0.001***	11.19	0.001**	3.57	0.009
Acidobacteria	2.17	0.079	0.40	0.529	0.42	0.828
Chloroflexi	38.79	<0.001***	3.98	0.053	2.18	0.077
Planctomycetes	15.12	<0.001***	0.01	0.931	0.65	0.661
Gemmatimonades	1.97	0.107	0.48	0.492	1.12	0.364
Firmicutes	6.43	<0.001***	11.95	0.001**	10.71	<0.001***
Bacteroidetes	6.77	<0.001***	0.12	0.722	0.98	0.441
Cyanobacteria	8.26	<0.001***	0.39	0.532	0.14	0.98
TM7	16.48	<0.001***	0.02	0.964	5.14	0.001**

TABLE S6. OTU Community assemblage analysis by PERMANOVA results based on 97% similarity OTU abundance data (square root transformed), using 999 permutations. Treatments consisted of control, microbial inoculant (microbes), mineral fertiliser (MF), and chemical fertiliser (CF) in different concentrations like 75kg ha^{-1} (CF-1), 55 kg ha^{-1} (CF-2) and 43kg ha^{-1} (CF-3). Significant *P* values indicated by * and *** corresponding to *P* < 0.05 and < 0.001, respectively.

	Degrees of freedom	Sum of Squares	Mean Squares	F. Model	R^2	Pr (>F)
Fertiliser	5	1.090	0.218	5.78	0.36	<0.001***
Harvest	1	0.106	0.106	2.82	0.03	0.003**
Fertiliser ×Hravest	5	0.461	0.092	2.44	0.15	<0.001***
Residuals	36	1.356	0.037	0.44		
Total	47	3.013	1.000			
